# No Association between Single Nucleotide Polymorphisms (SNPs) of the Interferon-Induced Transmembrane Protein 3 (*IFITM3*) Gene and the Susceptibility of Alzheimer’s Disease (AD)

**DOI:** 10.3390/medicina58010055

**Published:** 2021-12-30

**Authors:** Sae-Young Won, Yong-Chan Kim, Byung-Hoon Jeong

**Affiliations:** 1 Korea Zoonosis Research Institute, Jeonbuk National University, Iksan 570-390, Korea; gkfh32@jbnu.ac.kr (S.-Y.W.); kych@jbnu.ac.kr (Y.-C.K.); 2 Department of Bioactive Material Sciences, Jeonbuk National University, Jeonju 561-756, Korea

**Keywords:** Alzheimer’s disease, AD, IFITM3, SNP, polymorphism

## Abstract

*Background and Objectives*: Alzheimer’s disease (AD) is the most common progressive neurodegenerative disorder, characterized by the accumulation of amyloid-beta (Aβ) in the brain. A recent study reported that the interferon-induced transmembrane protein 3 (IFITM3) protein plays a pivotal role in Aβ processing by the γ-secretase complex. Since several single nucleotide polymorphisms (SNPs) of the *IFITM3* gene are related to the function and expression levels of the *IFITM3* gene, the relationship between genetic polymorphisms in the *IFITM3* gene and susceptibility to AD needs to be investigated. *Materials and Methods*: We investigated the genotype and allele frequencies of *IFITM3* polymorphisms in 177 AD patients and 233 matched healthy controls by amplicon sequencing. In addition, we compared the genotype, allele and haplotype frequencies between AD patients and matched controls and performed an association analysis. *Results*: There were no significant differences in the genotype, allele or haplotype frequency distributions of the *IFITM3* polymorphisms between AD patients and matched controls. *Conclusions*: To the best of our knowledge, this is the first case-control association study of the *IFITM3* gene in AD.

## 1. Introduction 

Alzheimer’s disease (AD) is the most common progressive neurodegenerative disease among the several types of dementia [1,2,3,4]. AD is characterized by the accumulation of amyloid beta-peptide (Aβ) derived from amyloid precursor protein (APP) in brain lesions. Aβ is produced by cleavage of the APP protein by the β-secretase encoded by the beta-site amyloid precursor protein cleaving enzyme 1 *(BASE1)* gene and γ-secretase, a complex that is comprised of a total of 4 high molecular weight subunits, including presenilin (PS), presenilin enhancer 2 (PEN-2), nicastrin and anterior pharynx defective 1 (APH-1) [5,6]. Several genetic variations of these genes affect the production of Aβ via the processing of APP protein. Thus, previous studies have reported that genetic variations in the *APP*, presenilin-1 (*PSEN1*) and presenilin-2 (*PSEN2*) genes are related to susceptibility to AD. In the *APP* gene, the genotype and allele frequencies of the -877T > C and -955A > G single nucleotide polymorphisms (SNPs) located in the promoter region were significantly associated with susceptibility to AD via transcription efficiency [7]. In addition, Q665D, K670M, N671L A692G, E693G, I716V and V717I germline mutations of the *APP* gene affect the onset of AD [8,9,10]. In the *PSEN1* and *PSEN2* genes, over 30 genetic variants are associated with elevated levels of Aβ40 and Aβ42 [8,9,10,11,12,13]. Based on this knowledge, several drugs targeting the APP and γ-secretase proteins have been introduced. However, there was no clear effect for any of the drugs tried [1,4]. Thus, it is necessary to discover specific targets to develop precise therapeutic agents for AD.

Interferon-induced transmembrane protein 3 (IFITM3) is a member of the innate immune system that is expressed primarily in the late endosome or lysosome and limits the entry of enveloped viruses [14,15,16,17,18,19,20]. In addition, a recent study have reported that upregulation of the IFITM3 protein affects γ-secretase, leading to increased Aβ production, and plays a pivotal role in the onset of AD [21]. In previous studies, the rs12252, rs34481144 and rs6598045 SNPs of the *IFITM3* gene were found to be involved in the gene expression level and function of the IFITM3 protein. The rs12252 C allele is related to cleavage of the 21 amino acids at the N-terminus of the IFITM3 protein and produces an isoform of the IFITM3 protein. The truncated IFITM3 protein induced by the rs12252 C allele has been shown to decrease the inhibition capacity of viral replication [16,22]. The A allele of the rs34481144 SNP inhibits the expression of the *IFITM3* gene by increasing the CCCTC-binding factor (CTCF) binding ability at the promoter site of the *IFITM3* gene [17]. The recent study has reported that rs12252 and 34481144 SNPs showed no correlation with the susceptibility of AD and the expression level of the IFITM3 protein. However, this study has performed association analysis with a relatively small sample size (control, n = 9; AD, n = 18) [21], and has not investigated rs6598045 SNP that modulates promoter activity [18]. Since several SNPs of the *IFITM3* gene are related to the function and expression level of the *IFITM3* gene and the IFITM3 protein plays a pivotal role in the processing of Aβ, further investigation of the relationship between genetic polymorphisms in *IFITM3* and susceptibility to AD is necessary.

In the present study, we investigated genotype, allele and haplotype frequencies of the *IFITM3* polymorphisms of 177 AD patients and 233 matched controls by amplicon sequencing. To find an association between *IFITM3* polymorphisms and susceptibility to AD, we compared genotype, allele, and haplotype frequencies of *IFITM3* polymorphisms between the two groups and performed association analysis.

## 2. Materials and Methods

### 2.1. Subjects

A total of blood samples from 177 AD patients were provided by the Pusan National University Hospital Biobank, a member of the Korea Biobank Network. A total of blood samples from 233 healthy Korean subjects were obtained from the Korea Biobank Network at the Centers for Disease Control and Prevention. The sample size used in this study may be enough to identify rare polymorphisms, including below 1% genotype frequency [22]. In addition, the sample size of healthy Korean subjects used in this study can also represent the total population of Korea with a 95% confidence level and a confidence interval of 6.42. The sample size of AD patients used in this study can also represent the total Korean AD patients with a 95% confidence level and a confidence interval of 7.36. All experimental procedures were approved according to the guidelines of the institutional review board of Jeonbuk National University and the 1964 Helsinki declaration and its later amendments or comparable ethical standards (approval number: JBNU 2019-03-009). Information for all samples was anonymized prior to investigation.

### 2.2. Genomic DNA Extraction

Genomic DNA was extracted from 200 µL of blood using the BeadTM Genomic DNA Prep Kit (Biofact, Daejeon, Korea) according to the manufacturer’s instructions.

### 2.3. Polymerase Chain Reaction (PCR)

The human *IFITM3* gene was amplified using PCR with the gene-specific forward primer: 5′-CAGGGGAAGTCTCCAGGACC-3′ and reverse primer: 5′-CCAAGCCACACACACACACA-3′. The PCR mixture consisted of 1 µL of genomic DNA, 10 pmol of each primer, 2.5 µL of 10× *Taq* DNA polymerase buffer, 0.5 µL of a 0.2 µM dNTP mixture, 5 µL of 5× Band Helper, and 0.25 µL *Taq* DNA polymerase. The PCR conditions followed the manufacturer’s recommendations. The annealing temperature of the *IFITM3* gene primers was 68 °C.

### 2.4. Amplicon Sequencing and Genotyping

All PCR products were purified using a FavorPrep GEL/PCR purification Mini Kit (FAVORGEN, Pingtung City, Taiwan) and sequenced on an ABI 3730 sequencer (ABI, Foster City, CA, USA). Sequencing results were visualized using Finch TV software (Geospiza Inc., Seattle, WA, USA), and genotyping was performed.

### 2.5. Statistical Analysis

The genotype and allele frequencies of the *IFITM3* gene were compared between the AD patients and healthy controls by the chi-square (χ^2^) test using SAS 9.4 software. Analysis of the Hardy-Weinberg equilibrium (HWE) test and haplotypes were performed using Haploview Version 4.2 (Broad Institute, Cambridge, MA, USA).

## 3. Results

### 3.1. Subject Description

A total of 177 Korean AD patients and 233 healthy Koreans without dementia were included in the association analysis. Detailed information on the study population is given in Table 1. The 177 AD patients included 128 females and 49 males. The 233 healthy individuals included 103 females and 130 males. The mean age of AD patients at diagnosis was 73.34 ± 7 years, and the mean age of healthy individuals at sample collection was 63.75 ± 9.74 years.

### 3.2. Evaluation of the Association of the Susceptibility of AD with the Genotype, Allele and Haplotype Frequencies of the IFITM3 Gene in the Korean Population

To investigate genotype and allele frequencies of the *IFITM3* gene, we performed direct sequencing in 233 healthy individuals and 177 AD patients and carried out genotyping and HWE analyses. Detailed information on the genotyping results and HWE values in the Korean population is described in Table 2.

**Figure 1:**
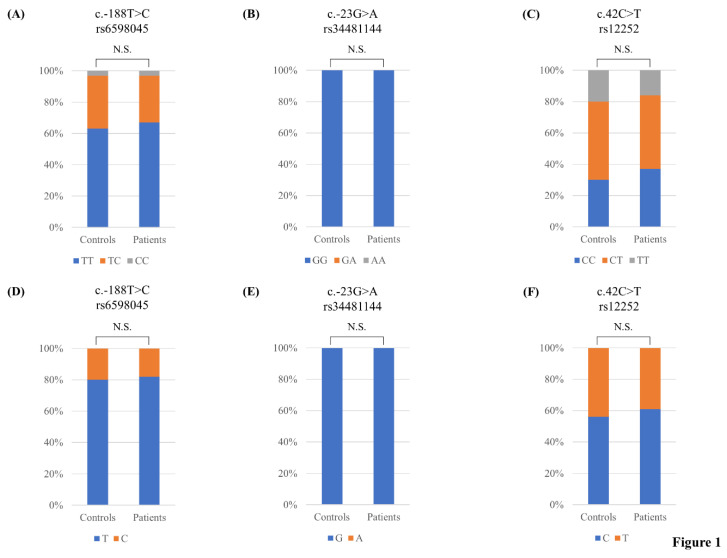
Comparisons of the genotype and allele frequencies of rs6598045, rs34481144 and rs12252 single nucleotide polymorphisms (SNPs) of the interferon-induced transmembrane protein 3 *(IFITM3)* gene in AD patients and matched healthy controls: (**A**) Comparison of the genotype frequency of the rs6598045 SNP of the *IFITM3* gene between these two groups; (**B**) Comparison of the genotype frequency of the rs34481144 SNP of the *IFITM3* gene between these two groups; (**C**) Comparison of the genotype frequency of the rs12252 SNP of the *IFITM3* gene in these two groups; (**D**) Comparison of the allele frequency of the rs6598045 SNP of the *IFITM3* gene between these two groups; (**E**) Comparison of the allele frequency of the rs34481144 SNP of the *IFITM3* gene between these two groups; (**F**) Comparison of the allele frequency of the *IFITM3* rs12252 SNP between these two groups. N.S.: not significant.

The genotype frequencies of 10 polymorphisms of the *IFITM3* gene were in HWE in the healthy controls. To estimate the association between susceptibility to AD and polymorphisms of the *IFITM3* gene, we performed case-control association analysis. There were no significant differences (*p* > 0.05) in the genotype or allele distributions of the *IFITM3* polymorphisms between healthy individuals and AD patients (Figure 1, Table 2).

Next, we performed haplotype analysis of 10 polymorphisms within the *IFITM3* gene, namely, c.-223C > G, c.-204G > T, c.-188T > C, c.-181T > C, c.-178A > C, c.-175T > C, c.-128C > T, c.-13_-4DelTTCGCTGGAC, c.42C > T and c.165C > T, in AD patients and healthy controls. All haplotypes showed similar distributions (*p* value > 0.5) in AD patients and healthy controls (Table 3).

## 4. Discussion

AD is characterized by the M1 type of disease-associated microglia (DAM) and the elevation of several neuroinflammation-related genes in the brain [23,24]. In a previous study, upregulation of the IFITM3 protein was also observed with neuroinflammation-related proteins by large-scale proteomic profiling in Aβ-treated microglia cell lines [25]. In addition, a recent study has reported that IFITM3 protein plays a major role in Aβ production via activation of γ-secretase [21]. Thus, the *IFITM3* gene was proposed as a novel therapeutic target for AD. A previous study did not find the association between AD and *IFITM3* SNPs. However, this study was performed with a relatively small sample size and, has not investigated the association of the rs6598045 SNP affecting the expression level of the *IFITM3* gene via TFII-I binding capacity [18,21]. Thus, we performed an association analysis of AD with *IFITM3* polymorphisms in larger populations. However, in the present study, we did not find significant differences in genotype, allele or haplotype frequencies of the *IFITM3* gene between AD patients and matched healthy controls (Table 2 and Table 3). In addition, when our data set were stratified by sex and *APOE* genotype, there were no differences in the genotype, allele or haplotype frequencies of the *IFITM3* gene between AD patients and matched controls (data not shown).

Previous studies have reported that the rs12252 C allele of the *IFITM3* gene was associated with the severity of infection for the influenza A H1N1 2009 pandemic virus [26,27,28,29]. In addition, it was suggested that the severity of influenza A 2009 virus infection varies among ethnic groups based on differences in the distribution of the *IFITM3* rs12252 SNP. The genotype and allele frequencies of rs12252, rs34481144, and rs6598045 SNPs are significantly different between Asian and Caucasian populations. In particular, although the risk of A allele of the rs34481144 SNP exists in less than 1% of East Asian individuals, the A allele of this SNP is present in a high proportion of individuals in European populations and is involved in the severity of pandemic influenza A 2009 virus infection [15,17,18]. Thus, in this study, although the SNPs of the *IFITM3* gene did not show an association with susceptibility to AD, the *IFITM3* SNPs is likely to affect AD phenotypes, including cognitive ability and Aβ accumulation. Thus, further investigation of the effect of the *IFITM3* SNPs on the phenotype of AD is needed. In addition, since this study investigated only a Korean population, further study is needed to validate the association between *IFITM3* SNPs and susceptibility to AD in other ethnic groups. Furthermore, since the genetic mechanism of the *IFITM3* gene was not related to susceptibility to AD, further investigation of the epigenetic mechanism of the *IFITM3* gene with AD is needed in the future.

## 5. Conclusions

In this study, we investigated genotype, allele, and haplotype frequencies of the *IFITM3* polymorphisms in AD patients and matched healthy controls. Notably, the genotype, allele, and haplotype distributions of the *IFITM3* polymorphisms did not show significant differences between these two groups. To the best of our knowledge, this is the first report regarding the association analysis of the *IFITM3* gene in AD patients.

## Figures and Tables

**Table 1:** Detailed information on the study population.

Characteristics		Cases	Controls
Number		177	233
Age		73.19 ± 7.20	63.75 ± 9.74
Sex (*n*, %)	Male	49	103
	Female	128	129
APOE	E2/E3	15 (8.5)	16 (7.1)
	E2/E4	7 (4)	1 (0.4)
	E3/E3	107 (60.5)	83 (37.2)
	E3/E4	39 (22)	12 (5.3)
	No data	9 (5)	111 (50)

**Table 2:** Comparison of genotype and allele frequencies between healthy controls and Alzheimer’s disease (AD) patients in a Korean population.

Variants		Genotype Frequency, *n* (%)			*p*-Value	Allele Frequency, *n* (%)		*p*-Value	HWE
		CC	CG	GG		C	G		
c.-223C > G	Controls	232 (1)	1 (0)	0 (0)		465 (1)	1 (0)		0.9738
Rs1297064916	Patients	177 (1)	0 (0)	0 (0)	1.0	354 (1)	0 (0)	1.0	-
		GG	GT	TT		G	T		
c.-204G > T	Controls	91 (0.39)	109 (0.47)	33 (0.14)		291 (0.62)	175 (0.38)		0.9686
Rs3888188	Patients	78 (0.44)	78 (0.44)	21 (0.12)	0.5546	234 (0.66)	120 (0.34)	0.2800	0.8245
		TT	TC	CC		T	C		
c.-188T > C	Controls	146 (0.63)	80 (0.34)	7 (0.03)		372 (0.80)	94 (0.2)		0.3129
Rs6598045	Patients	118 (0.67)	53 (0.3)	6 (0.03)	0.6390	289 (0.82)	65 (0.18)	0.5161	0.9870
		TT	TC	CC		T	C		
c.-181T > C	Controls	135 (0.58)	84 (0.36)	14 (0.06)		354 (0.76)	112 (0.24)		0.8461
Rs7478728	Patients	118 (0.67)	48 (0.27)	11 (0.6)	0.1540	284 (0.8)	70 (0.2)	0.1459	0.0532
		AA	AC	CC		A	C		
c.-178A > C	Controls	136 (0.58)	83 (0.36)	14 (0.06)		355 (0.76)	111 (0.24)		0.7782
Rs71452596	Patients	118 (0.67)	48 (0.27)	11 (0.06)	0.1826	284 (0.8)	70 (0.2)	0.1665	0.0532
		TT	TC	CC		T	C		
c.-175T > C	Controls	136 (0.59)	82 (0.35)	14 (0.06)		354 (0.76)	110 (0.24)		0.7271
Rs7479267	Patients	118 (0.67)	48 (0.17)	11 (0.06)	0.2030	284 (0.8)	70 (0.2)	0.1785	0.0532
		CC	CT	TT		C	T		
c.-128C > T	Controls	229 (0.98)	4 (0.02)	0 (0)		462 (0.99)	4 (0.01)		0.8949
Rs536077498	Patients	173 (0.98)	4 (0.02)	0 (0)	0.7307	350 (0.99)	4 (0.01)	0.7318	0.8792
		GG	GA	AA		G	A		
c.-23G > A	Controls	233 (1)	0 (0)	0 (0)		466 (1)	0 (0)		-
Rs34481144	Patients	177 (1)	0 (0)	0 (0)	-	354 (1)	0 (0)	-	-
		Wt/Wt	Wt/Del	Del/Del		Wt	Del		
c.-13_-4Del	Controls	232 (1)	1 (1)	0 (0)		465 (1)	1 (0)		0.9738
TTCGCTGGAC	Patients	177 (1)	0 (0)	0 (0)	1.0	354 (1)	0 (0)	1.0	-
Rs773536963									
		CC	CT	TT		C	T		
c.42C > T	Controls	73 (0.3)	116 (0.5)	44 (0.2)		262 (0.56)	204 (0.44)		0.8621
Rs12252	Patients	66 (0.37)	83 (0.47)	28 (0.16)	0.4138	215 (0.61)	139 (0.39)	0.1946	0.8228
		CC	CT	TT		C	T		
c.165C > T	Controls	231 (0.99)	2 (0.01)	0 (0)		464 (1)	2 (0)		0.9475
Rs11553885	Patients	175 (0.99)	2 (0.01)	0 (0)	1.0	352 (1)	2 (0)	1.0	0.9397

**Table 3:** Haplotype frequencies of *IFITM3* polymorphisms in the healthy controls and Alzheimer’s disease (AD) patients.

Haplotype	Frequency		*p*-Value
	Control (*n* = 354)	Patients (*n* = 466)	
CGTTATCwtCC	193 (0.545)	272 (0.583)	0.2705
CTTCCCCwtTC	82 (0.232)	90 (0.193)	0.1798
CTCTATCwtTC	49 (0.139)	63 (0.135)	0.8940
CGCTATCwtTC	20 (0.056)	21 (0.045)	0.4569
Others	10 (0.028)	20 (0.044)	0.2677

## Data Availability

All data are available from the corresponding authors upon reasonable request.
